# Effects of immunotherapy induction on outcome and graft survival of kidney-transplanted patients with different immunological risk of rejection

**DOI:** 10.1186/s12882-019-1497-5

**Published:** 2019-08-13

**Authors:** Marcus Faria Lasmar, Rodrigo Santana Dutra, José Augusto Nogueira-Machado, Raquel A. Fabreti-Oliveira, Raquel Gomes Siqueira, Evaldo Nascimento

**Affiliations:** 1 University Hospital of the Faculty of Medical Science, Belo Horizonte, Minas Gerais state Brazil; 2 Institute of Research and Education of the Hospital Santa Casa, Belo Horizonte, Minas Gerais state Brazil; 3 Faculty of Medical Sciences, Belo Horizonte, Minas Gerais State Brazil; 4 IMUNOLAB – Laboratory of Histocompatibility, Minas Gerais state, Belo Horizonte, Brazil

**Keywords:** Kidney transplantation, Outcome, Thymoglobulin, Immunotherapy induction, Risk of rejection, Graft survival

## Abstract

**Background:**

In kidney transplantation, immunotherapy with thymoglobulin (rATG) has been used to down-regulate the patient immune system. rATG is a powerful immunobiologic drug used to deplete lymphocytes to prevent early acute rejection. The aim of this research was to evaluate the effects of immunotherapy by rATG on graft suvival during a 9-year period in kidney-transplanted patients with different immunological profiles.

**Methods:**

A sample of 469 patients were allocated into four groups (G) based on immunological risk of rejection: G1, low risk, not sensitized recipients, solid-phase immunoassay with single antigen beads (SPI-SAB) < 10%; G2, medium risk I, sensitized recipients, SPI-SAB ≥ 10 < 50%; G3, medium risk II sensitized (SPI-SAB ≥50%); and G4, high risk, sensitized recipients, SPI-SAB- donor-specific antibody positive (DSA+). Only patients from G3 and G4 received immunotherapy.

**Results:**

Of 255 patients who received a kidney from a living donor (LD), 42 (16.47%) from all groups (G) had T-cell–mediated rejection (TCMR) and four (G1) lost their grafts, 8 (3.14%) had antibody-mediated rejection (AMR), and two lost their graft in G1 and G4. Of 214 patients who received a kidney from deceased donors (DD), 37 (17.29%) had TCMR with one lost graft in G1. AMR was shown in 13 (6.07%) patients, with three losses observed in G2. Statistical differences between the groups in the 9-year graft survival rate were found only in the comparison of G1 versus G2 (*P* = 0.005) and G2 versus G4 (*P* = 0.047) for DD. For LD, no statistical differences were found.

**Conclusion:**

This clinical retrospective study shows that immunotherapy induction was associated with improvement of outcomes, graft function, and survival in patients treated with immunotherapy in comparison with patients who did not received induction therapy. These findings strongly suggest that immunotherapy should be used for all patients transplanted with kidneys from deceased donors.

## Background

Immunotherapy induction has been used for decades in patients with chronic kidney disease (CKD) to accept allografts and to obtain durable kidney graft survival. In recent years, the surgical procedure has become simple and easy to perform, and the use of short-term immunotherapy with immunobiologic drugs in the perioperative period to prevent acute graft rejection has become common [1, 2]. The immunobiologic thymoglobulin (rATG) became available at least 20 years ago as a rabbit-derived polyclonal antibody, and it has been used for immunotherapy in kidney transplant for providing suitable long-term graft outcome [3]. Studies have shown that rATG is a powerful drug widely prescribed with the benefit of low cost in comparison with other rejection therapies. The mechanism by which rATG induces immunosuppression includes T-cell subset depletion and different methods of modulation, such as Fc-receptor mediated complement-dependent lysis, opsonization and phagocytosis by macrophages, and immunomodulation. These processes lead to long-term depletion via apoptosis and antibody-dependent T-cell cytotoxicity to produce effective immunoregulation of the patient’s immune system, which allows tolerance of donor human leukocyte antigen (HLA) mismatches to avoid early rejection episodes after kidney transplantation [4–18]. The goal of immunotherapy is to induce tolerance, allow for a minimal dose of maintenance immunosuppressive drugs to prevent toxicity effects in the patient, and, primarily, to achieve a long-term graft outcome [19–21].

In recent years, classifying kidney-transplanted patients by immunological risk using anti-HLA–specific antibody identified before transplantation has been used with great benefit for graft outcome [22]. Currently, patients are classified as at low, medium or intermediate, and high immunological risk for antibody-mediated rejection (AMR) [23–25].

Many studies seeking to improve long-term graft survival have described the beneficial effects linked to HLA matching between recipient and donor, immunological receptor profile, duration of cold ischemia time (CIT), and immunotherapy induction to provide durable kidney function. In most patients, graft failure is caused by antibodies directed against a wide range of shared mismatching epitopes in HLA molecules in recipient and donor [3, 26–28], donor-specific antibody (DSA), estimation of rejection risk, presence of delayed graft function (DGF), patient care, donor age, and triple-drug immunosuppression therapy [29]. The half-life of graft survival remains approximately 12 years [30]. However, graft survival from living and deceased donors of more than 20 years was demonstrated in transplanted recipients treated with azathioprine monotherapy [31], and long-term graft survival of more than 30 years was also similar between grafts from both types of donors [32]. A recent ultra-long-term graft survival using monotherapy was demonstrated in a patient who lived 42 years with a 100-year-old graft received from a related living donor [33]. The aim of this study was to evaluate the effects of immunotherapy by rATG on graft suvival during a 9-year period in kidney-transplanted patients with different immunological profiles.

## Methods

### Patient population

This retrospective study was conducted using a nonrandomized convenience sample of recipients transplanted for the first time with kidneys from living donors (LD) or deceased donors (DD) from 2008 to 2016. All immunological tests before transplantation, such as HLA typing, solid-phase immunoassay–single antigen beads (SPI-SAB) using a 100IS fluoroanalyzer (Luminex Inc., Austin, TX, USA), and crossmatches for B and T lymphocytes [26] were performed by IMUNOLAB–Laboratory of Histocompatibility, Belo Horizonte, Minas Gerais, Brazil. Antibody specificities with a mean fluorescence intensity (MFI) of ≥500 were considered positive. All patients were allocated into four groups (G) by immunological profile: G1, low risk, not sensitized recipients, SPI-SAB < 10%; G2, medium risk I, sensitized recipients, SPI-SAB ≥ 10 < 50%; G3, medium risk II sensitized (SPI-SAB ≥50%); and G4, high risk, sensitized recipients, SPI-SAB-DSA+. Surgery was performed at the Transplantation Center of the University Hospital of the Faculty of Medical Science, Belo Horizonte, Minas Gerais state, Brazil. Patients from G3 received immunotherapy induction with rATG. The diagnosis of rejection type was graded according to the Banff 2013 and 2015 classification [34, 35].

### Immunotherapy induction

Immunotherapy induction with rATG (Thymoglobulin, Genzyme, Mississauga, Canada) was started intraoperatively after the patient was anesthetized and was stopped 4 h later. The beginning dose was 1 mg/kg until 4.5 mg/kg. In the following 7 days after transplantation, the doses of rATG were administered taking into account the number of blood lymphocytes and platelets. Patients were treated and monitored daily using the parameter of > 300 lymphocytes/mm^3^ and > 5 × 10^4^ platelets/mm^3^. The rATG concentration was reduced to 1 mg/kg when the cell count was < 300 cells/mm^3^, and patients with < 5 × 10^4^ platelets/mm^3^ received 0.5 mg/kg. rATG was temporarily suspended or interrupted if severe adverse events such as anaphylaxis, pulmonary edema, malignancies, and virus infections were detected clinically or through laboratory testing [36].

### Maintenance immunosuppressive drugs (MISD)

After immunotherapy, MISD therapy using a three-drug regimen, such as 0.25 to 0.3 mg/kg tacrolimus (TAC; Libbs Laboratory, São Paulo, Brazil), was initiated, and a target whole-blood level of 10 to 12 ng/mL in the first month, 8 to 10 ng/mL in the second month, and 5 ng/mL in the following months was considered effective for immunosuppressive activity. In the regimen using cyclosporine A (CyA; Biosintética, São Paulo, Brazil) the dose was 5 to 8 mg/kg/day, and the blood concentration was 250 to 300 ng/mL in the first month, 200 to 250 ng/mL in the second month, and 150 to 200 ng/mL in the following months. The measurement of whole-blood drug concentration was performed by Hermis Pardini Laboratory (Belo Horizonte, MG, Brazil). In addition to these drugs, 0.5 mg/kg of the corticosteroid methylprednisone was administered orally (Methicorten, Sheering-Plough, São Paulo, Brazil) in the first 2 months and 5 mg/day in the following months after transplantation in addition to sodium mycophenolate (MPA; Novartis, Basel, Switzerland) at 720 mg twice daily.

This maintenance protocol was adjusted for patients who had side effects of calcineurin inhibitors proved by biopsy, diarrhea and abdominal pain, weight loss, or skin cancer or when virus reinfection by cytomegalovirus, polyomavirus, or human papilloma virus was detected. These patients were converted to MPA therapy with azathioprine (Aspen Pharma, Serra/ES, Brazil) at a dosage of 2 mg/kg/day.

Patients with CyA or TAC nephrotoxicity proved by biopsy were converted to sirolimus therapy (Pfizer, São Paulo, Brazil) at a dosage of 1 to 4 mg/day with serum concentrations of 6 to 12 μg/mL or to everolimus (Novartis, Basileia, Swiss) at a dosage of 1 to 4 mg/day with serum concentrations of 3 to 8 μg/mL. All patient blood samples were taken 30 min before the next medications were administered. Drug concentration was analyzed by the Hospital of Kidney and Hypertension Laboratory, São Paulo, Brazil. Corticoid methylprednisone was discontinued only in patients with head femur necrosis.

### Rescue therapy

Patients with clinical symptoms of graft rejection were biopsied in the hospital transplantation center, and blood was collected to perform SPI-SAB. Patients with cellular rejection classified according BANF criteria (34,35) or cases of borderline IA, IB, and IIA were treated with methylprednisone at dosages of 500 to 1000 mg/day for 3 days. Patients with corticosteroid-resistant rejection classified as IIB or III were treated by immunotherapy with 7 mg/kg rATG for 5 to 7 days. Patients with AMR were diagnosed with C4d + by histology, and DSA+ patients were treated using a combination of plasmapheresis, MPA, and immunotherapy with 7 mg/kg of rATG for 5 to 7 days adjusted according to the minimum level of leukocytes and platelets. Reversal of rejection was defined as a return of serum creatinine within 20 days after antirejection therapy. All immunosuppressive drugs were provided to patients by the Brazilian Public Health System.

### Statistical analysis

Statistical analysis of the comparison between groups was performed using a database containing all the information from patients and donors. The analysis was performed using SPSS software version 18.0 (Chicago, IL, USA). Differences were considered statistically significant if *P* < 0.05. The Kolmogorov-Smirnov normality test was performed for all continuous numerical variables. For variables with normal distributions, the means were compared using Student *t-*test or one-way analysis of variance by *F* test, whereas for variables with no normal distributions, the comparisons were made using the Kruskal-Wallis nonparametric test. The chi-square of Pearson, Fisher’s exact, and likelihood ratio tests were used to compare categorical variables. Graft survival analysis was performed using the Kaplan-Meier method and log-rank tests for group comparisons. Proportional hazards was used as the model for the Cox regression survival analysis. Based on the significant variables (*P* ≤ 0.20) in the univariate analysis, the model was constructed by the stepwise backward method. The covariates included in the multivariate analysis were recipient and donor age, immunological profile, CIT, and change in the triple-drug maintenance therapy (TDMT). For permanence of the variables in the final model, a significance level of 0.05 was considered significant. The importance of each covariant in the model was assessed by the Wald test. We used Schoenfeld waste for the evaluation of the assumption of proportionality of the risks.

## Results

Statistically significant differences between genders were observed in groups who received kidneys from both type of donors (*P* < 0.001; Table 1). These differences can be ascribed to the presence of more males in G1 or G2 than females in comparison with patients from G3 and G4. Considering donor type, no statistical differences were observed in patient age between the groups nor between patients classified by ABO blood type (Table 1). However, the recipient age was higher in those who received grafts from DD than from LD, 48.58 and 41.62 years, respectively (*P* < 0.001). In addition, no statistically significant differences were observed in the comparison of the proportion of patients by etiological causes of CKD (Table 1). In the comparison between type of dialysis therapies or preemptive patients, no statistically significant differences were found between the groups of transplanted patients with LD and DD. However, in patients who received a kidney from LD, significant statistical differences were found for the time patients spent in dialysis therapy (*P* = 0.001). The time spent in dialysis was longer for patients transplanted with a kidney from a DD (55.45 ± 38.73 months) as compared with from an LD (25.14 ± 29.68 months; *P* < 0.001). For recipients who received a kidney from a DD, there were no statistically significant differences found for CIT, but significance was found for expanded criteria (*P* = 0.014; Table 1).

**Table 1:** Demographic and clinical data of patients by type of donor and risk groups for antibody-mediated rejection

Variable	LD (*n* = 255)					DD (*n* = 214)				
	G1	G2	G3	G4	*P* value	G1	G2	G3	G4	*P* value
Number of patients	191 (74.90%)	41 (16.08%)	12 (4.71%)	11 (4.31%)		150 (70.09%)	34 (15.89%)	14 (6.54%)	16 (7.48%)	
Gender										
*Male*	143 (74.87%)	13 (31.71%)	1 (8.33%)	4 (36.36%)	**< 0.001**	111 (74.00%)	25 (73.53%)	3 (21.43%)	7 (43.75%)	**< 0.001**
*Female*	48 (25.13%)	28 (68.29%)	11 (91.67%)	7 (63.64%)		39 (26.00%)	9 (26.47%)	11 (78.57%)	9 (56.25%)	
Age (years) ± SDV	41.49 ± 11.58	41.88 ± 11.45	38.33 ± 8.283	46.45 ± 12.396	0.396	48.50 ± 11.83	51.38 ± 14.67	45.64 ± 15.16	45.94 ± 13.68	0.377
ABO group (*n* = 467)										
*O*	94 (49.47%)	24 (58.54%)	5 (41.67%)	6 (54.55%)	0.559	68 (45.64%)	15 (44.12%)	5 (35.71%)	10 (62.50%)	0.891
*A*	69 (36.32%)	12 (29.27%)	7 (58.33%)	3 (27.27%)		57 (38.26%)	13 (38.24%)	6 (42.86%)	3 (18.75%)	
*B*	23 (12.11%)	4 (9.76%)	0 (0%)	1 (9.09%)		11 (7.38%)	3 (8.82%)	2 (14.29%)	2 (12.50%)	
*AB*	4 (2.11%)	1 (2.44%)	0 (0%)	1 (9.09%)		13 (8.72%)	3 (8.82%)	1 (7.14%)	1 (6.25%)	
Etiology of CKD										
*Hypertensive nephropathy*	16 (8.38%)	8 (19.51%)	1 (8.33%)	2 (18.18%)	0.616	28 (18.67%)	6 (17.65%)	2 (14.29%)	1 (6.25%)	0.246
*Diabetes mellitus*	21 (10.99%)	3 (7.32%)	0 (0%)	1 (9.09%)		25 (16.67%)	4 (11.76%)	2 (14.29%)	3 (18.75%)	
*Glomerulopathy*	45 (23.56%)	9 (21.95%)	3 (25.00%)	1 (9.09%)		23 (15.33%)	9 (26.47%)	1 (7.14%)	1 (6.25%)	
*Autosomal polycystic kidney disease*	18 (9.42%)	3 (7.32%)	1 (8.33%)	1 (9.09%)		17 (11.33%)	3 (8.82%)	2 (14.29%)	1 (6.25%)	
*Undetermined*	83 (43.46%)	15 (36.59%)	5 (41.67%)	6 (54.55%)		54 (36.00%)	8 (23.53%)	7 (50.00%)	8 (50.00%)	
*Others*	8 (4.19%)	3 (7.32%)	2 (16.67%)	0 (0%)		3 (2.00%)	4 (11.76%)	0 (0%)	2 (12.50%)	
Type of dialysis										
*Hemodialysis*	148 (77.49%)	36 (87.80%)	11 (91.67%)	11 (100.00%)	0.141	142 (94.67%)	32 (94.12%)	12 (85.71%)	16 (100.0%)	0.672
*Peritoneal dialysis*	14 (7.33%)	1 (2.44%)	0 (0%)	0 (0%)		6 (4.00%)	1 (2.94%)	1 (7.14%)	0 (0%)	
*Preemptive*	29 (15.18%)	4 (9.76%)	1 (8.33%)	0 (0%)		2 (1.33%)	1 (2.94%)	1 (7.14%)	0 (0%)	
Time in dialysis (mo) ± SDV	21.09 ± 24.12	34.93 ± 34.07	51.25 ± 65.06	30.45 ± 21.36	**< 0.001**	55.29 ± 40.13	52.91 ± 34.64	52.57 ± 27.28	64.88 ± 43.58	0.762
DGF										
*Yes*	–	–	–		–	94 (62.67%)	18 (52.94%)	6 (42.86%)	12 (75.00%)	0.227
*No*	–	–	–			56 (37.33%)	16 (47.06%)	8 (57.14%)	4 (25.00%)	
Cold ischemia time (h) ± SDV	–	–	–		–	16.74 ± 5.57	17.28 ± 6.72	14.91 ± 4.39	19.04 ± 5.72	0.272
Expanded criteria	–	–	–							
*Yes*	–	–	–		–	36 (24.00%)	3 (8.82%)	1 (7.14%)	7 (43.75%)	**0.014**
*No*	–	–	–			114 (76.00%)	31 (91.18%)	13 (92.86%)	9 (56.25%)	

*CKD* chronic kidney disease, *DGF* delayed graft function

*P*-values in boldface are statistically significant

In the 255 patients who received a kidney from a LD, 42 (16.47%) had rejection by T-cell–mediated rejection (TCMR). Of these, 31, 8, 3, and 2 patients with TCMR were from groups G1, G2, G3, and G4, respectively (Table 2). Of these, 4 (2.09%) from G1 lost their grafts. On the other hand, 8 patients had AMR. Of these, 2 (0.78%) patients lost their grafts: one in G1 and another in G4 due to HLA class II DSA DR7 (MFI = 2.170) and DR9 (MFI = 4.081). In G4, 1 graft loss was observed due to de-novo HLA class I DSA B18 (MFI = 1.342; Table 2), but in this patient, DSA was not reported before transplantation. In the patients from G2 that received kidney from DD two grafts were lost due to HLA class I DSA and one due to HLA class I and II DSAs (Table 2). Graft lost without immunological cause was mainly due to infections and obits. The data also demonstrated no significant differences in the serum creatinine level (Table 2). Moreover, there were no differences in HLA compatibility observed among the groups (*P* = 0.128).

**Table 2:** Nine-year outcomes among 469 patients with different immunological risks transplanted with kidneys from living (LD) and deceased donors (DD)

Variable	LD (*n* = 255)					DD (*n* = 214)				
	G1 (*n* = 191)	G2 (*n* = 41)	G3 (*n* = 12)	G4 (*n* = 11)	Total	G1 (*n* = 150)	G2 (*n* = 34)	G3 (*n* = 14)	G4 (*n* = 16)	Total
Rejection episodes										
TCMR	31 (16.23%)	8 (19.51%)	2 (16.67%)	1 (9.09%)	42 (16.47%)	30 (20.00%)	5 (14.71%)	2 (14.29%)	0	37 (17.29%)
AMR	4 (2.094%)	1 (2.44%)	1 (8.34%)	2 (18.18%)	8 (3.14%)	7 (4.67%)	3 (8.82%)	2 (14.29%)	1 (6.25%)	13 (6.07%)
TCMR/AMR	0	0	0		0	1 (0.67%)	1 (2.94%)	0	0	2 (0.93%)
Graft loss										
TCMR	4 (2.09%)	0	0	0	4 (1.57%)	1 (0.67%)	0	0	0	1 (0.47%)
AMR	1 (0.53%) ID359: DR7 (2170); DR9 (4081)	0	0	1 (9.09%) ID363: B18 (1342)	2 (0.78%)	0	3 (8.82%)	0	0	3 (1.40%)
							ID97: A11 (6612); B44 (15026)			
							ID233: A1 (815); B52 (1507)			
							ID290: A23 (1906); B65 (1078); DR1 (1614)			
Other causes of graft loss										
Cardiovascular	4 (2.09%)	0	0	0	0	1 (0.67%)	0	0	0	1 (0.47%)
Infections	12 (6.28%%)	1 (2.44)	0	0	5 (1.96%)	9 (6.00%)	2 (5.88%)	0	1 (6.25%)	4 (1.87%)
Death	21 (10.99%)	2 (4.88%)	1 (8.33%)	0	20 (7.84%)	20 (13.33%)	10 (29.42%)	2 (14.29%)	3 (18.75%)	32 (14.95%)
Other	22 (11.52%)	4 (9.76%)	0	0	22 (8.63%)	18 (12.0%)	4 (11.76%)	1 (7.14%)	0	19 (8.88%)
HLA mismatching										
0	34 (17.80%)	8 (19.51%)	4 (33.33%)	0 (0%)	0.128	2 (1.33%)	0	0 (0%)	0 (0%)	0.053
1–3	90 (47.12%)	21 (51.22%)	6 (50.00%)	9 (81.82%)		91 (60.67%)	23 (67.65%)	11 (78.57%)	4 (25.00%)	
4–6	67 (35.08%)	12 (29.27%)	2 (16.67%)	2 (18.18%)		57 (38.00%)	11 (32.35%)	3 (21.43%)	12 (75.00%)	
Creatinine (mean ± SD)										
Months										
1	1.72 ± 1.16	1.44 ± 0.73	1.65 ± 1.42	1.42 ± 0.40	0.468	2.39 ± 1.66	2.44 ± 1.49	2.28 ± 1.88	2.74 ± 1.43	0.854
3	1.63 ± 1.00	1.28 ± 0.40	1.35 ± 0.41	1.37 ± 0.30	0.107	1.99 ± 1.30	1.86 ± 1.13	1.57 ± 0.62	2.34 ± 1.13	0.411
6	1.58 ± 0.87	1.24 ± 0.37	1.28 ± 0.27	1.32 ± 0.30	0.066	1.75 ± 0.89	2.06 ± 1.94	1.53 ± 0.44	2.09 ± 0.96	0.320
Years										
1	1.52 ± 0.76	1.33 ± 0.57	1.21 ± 0.27	1.29 ± 0.49	0.215	1.73 ± 0.93	1.56 ± 0.67	1.42 ± 0.68	1.72 ± 0.57	0.610
3	1.45 ± 0.55	1.41 ± 0.68	1.13 ± 0.24	1.73 ± 1.42	0.273	1.72 ± 1.18	1.73 ± 1.04	1.41 ± 0.70	1.64 ± 0.40	0.907
5	1.54 ± 0.83	1.46 ± 0.55	0.99 ± 0.20	1.12 ± 0.34	0.329	1.53 ± 0.77	1.30 ± 0.45	1.25 ± 0.27	1.63 ± 0.46	0.735
7	1.25 ± 0.50	1.52 ± 0.58	1.05 ± 0.37	0.94 ± 0.72	0.427	–	–	–	–	–
9	–	–	–	–	–	–	–	–	–	–

From the 214 patients from G1, G2, G3, and G4 who received a kidney from a DD, 37 (17.29%) patients had TCMR, and only 1 lost the graft in G1 (Table 2). Episodes of rejections by AMR were demonstrated in 13 (6.07%) patients from these groups. In G2, 3 patients had rejection episodes, and 3 lost their grafts. In the first case, there was HLA class I DSA A11 (MFI = 6.612) and B44 (MFI = 15.026). In the second case, patients had TCMR in the first months and lost their graft 15 months after transplant by AMR due to HLA class I DSA-A1 (MFI = 815) and B52 (MFI = 1.507), and the third patient lost the graft by HLA class I DSA-A23 (MFI = 1.906), B65 (MFI = 1.078), HLA class II DR1 (MFI = 1.614) (Table 2). Loss was not shown in G3 or G4 by TCMR or AMR. The main non immunological causes involved in graft loss were infections and death. In the patients who received a kidney from a DD, no statistically significant differences were observed in HLA compatibility or creatinine levels (Table 2).

Among those who received a kidney from an LD, the number of patients with a functioning graft was 127 (66.49%), 34 (82.93%), 11 (91.67%), and 10 (90.91%) from G1, G2, G3, and G4, respectively. For those transplanted with a kidney from a DD, the number of patients with a functional graft was 101 (67.33%), 15 (44.12%), 11 (78.57%), and 12 (75.0%) in G1, G2, G3, and G4, respectively (Table 2). Table 3 shows that the risk of graft loss for sensitized patients with medium risk I (G2) without immunotherapy was 1.845 (1.093–3.661) times that of patients in G1 (*P* = 0.028). Patients who had TDMT conversion had almost a 50% less risk for graft loss in comparison to those without conversion. These data showed that the age of the DD, chance of triple-drug maintenance immunosuppression, and immunotherapy were found to be predictive factors for graft loss (Table 3). The age of the recipient and CIT variables were present in the multivariate Cox analysis but were not significant and therefore excluded from the final model by the backward Wald method.

**Table 3:** Hazard rates for predictive variables of risk for graft loss in transplanted patients with a kidney from a deceased donor

Predictive variable	Hazard rate (CI = 95%)	*P* value
Age of donor	1.032 (1.006–1.058)	**0.016**
Change in the TDMI	0.519 (0.271–0.981)	**0.047**
Immunological profile:		
G1	–	–
G2	1.845 (1.093–3.661)	**0.028**
G3^a^	1.454 (0.336–3.287)	0.617
G4	0.667 (0.199–2.240)	0.513

*CI* confidence interval, *G* group, *TDMI* triple-drug maintenance immunosuppression. ^a^Immunotherapy

*P*-values in boldface are statistically significant

Statistical differences in the 9-year graft survival rate between the groups were found only in the comparisons of G1 versus G2 (*P* = 0.005) and G2 versus G4 (*P* = 0.047).

**Fig. 1:**
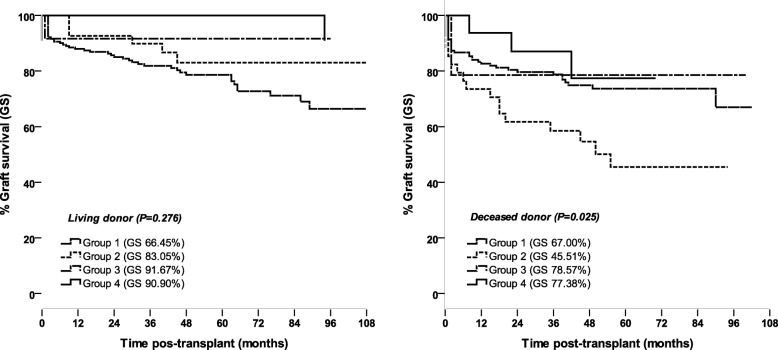
Analysis of graft survival by Kaplan-Meier method in groups of patients with different immunological risks of antibody-mediated rejection. **a**: living donor. **b**: deceased donor. G1: low risk, not sensitized recipients, solid-phase immunoassay with single antigen beads (SPI-SAB) < 10%; G2: medium risk I, sensitized recipients, SPI-SAB ≥ 10 < 50%; G3: medium risk II sensitized (SPI-SAB ≥50%); G4: high-risk, sensitized recipients, SPI-SAB-DSA+. For patients who received DD, statically significant differences were found only in the comparison between G1 versus G2 (*P* = 0.005) and G2 versus G4 (*P* = 0.047)

The 9-year graft survival rates for LD-transplanted patients were 66.45% for patients with no immunological risk from G1, 83.05% for sensitized patients at medium risk I from G2, 91.67% for sensitized patients at medium risk II from G3, and 90.90% for high-risk patients from G4. No statistical differences in survival (*P* = 0.276) were observed among the groups, although G3 and G4 had a trend toward better outcome and graft survival than G1 and G2. For transplanted patients with a kidney from a DD, survival rates of 67.0, 45.51, 78.57, and 77.38% for G1, G2, G3, and G4 were found, respectively. There were statistically significant differences in survival only in the comparison of G1 versus G2 (*P* = 0.005) and G2 versus G4 (*P* = 0.047) (*P* = 0.025), with G3 or G4 presenting better and G2 worse survival rates (Fig. 1).

## Discussion

This study showed better outcome and graft survival rates in patients who received immunotherapy compared with those who did not. Traditionally, recipients of kidneys from an LD are presumed to be at lower risk for rejection than those receiving a kidney from a DD. However, we observed a high incidence of TCMR in LD recipients due in part to induction therapy in this population. The importance of antibody induction was highlighted in an analysis showing that acute rejection was the most significant factor affecting long-term outcomes in LD recipients, whereas outcomes in DD transplants were dependent on both immumologic and nonimmunologic factors [37]. Use of rabbit antithymocyte (rATG) in LD transplantation has recently increased to more than 40%; this increase may be related to the desire to further reduce the incidence of reinfection, allowing for steroid withdrawal and minimizing the exposure to calcineurin inhibitors [38]. Moreover, for G3 and G4 with hypersensitized patients with or without DSA, the risk of graft loss was similar to the nonsensitized patients, probably because of immunotherapy. We found a greater number of male recipients, which may be associated with the predominance of dialysis in men among patients with chronic renal failure [39]. However, the proportion of females was higher in sensitized groups. The frequency of kidney transplants from an LD (54.37%) was higher than from a DD (45.63%), which may be a specific characteristic of this university hospital in relation to other transplantation centers.

A higher frequency of DGF was observed for all groups of patients transplanted with a kidney from a DD, with a general proportion of 60.75%. This finding may be related to the long length of CIT for DD (about 17 h). The CIT was a predictive factor for the development of DGF; the risk of DGF was 2.45 times higher for transplant recipients with a CIT > 12 h [29].

Graft survival at 9 years for patients who received a kidney transplant from an LD was 66.45% in G1, 83.06% in G2, 91.67% in G3, and 90.90% in G4. In the DD group, it was 67.0, 45.51, 78.57, and 77.38% in patients from G1, G2, G3, and G4, respectively. However, in the global analysis, the graft survival rates were 71.40 and 63.80% for the LD and DD groups, respectively. Our data were quite similar to United Network for Organ Sharing data records of 2016, which found 82.00% for LD and 57.20% for DD for 10-year outcome.

Over 9 years of follow-up, the survival rate was similar in G1 for LD and DD, although a better outcome was expected for LD [40]. In this study, considering all causes, we observed a greater proportion of graft losses in G1 with a kidney from an LD mainly due to TCMR (16.23%) and infection (11.11%) compared with 20.0 and 8.11%, respectively, for patients from G1 transplanted with a kidney from a DD. The complications due to infectious diseases observed in our study were an important cause of death differing from other data, in which infectious diseases were the second leading cause of death, behind complications from cardiovascular diseases [41].

The synthesis of anti-HLA antibodies before and after transplantation has been considered to be an important risk factor for graft outcomes, and knowledge of the role of DSA in organ transplantation has improved significantly over the past decade [42–44]. Because of the development of more sensitive solid-phase immunoassays for identified DSA, it has become one of the most important biomarkers of a risk factor for injury and graft loss [27, 43]. The data obtained in this study showed that the patient population was comparable with that of a multicenter study perfomed using similar immunosupressive regimens [43].

The stratification of patients by immunological risk and type of donor facilitated patient care, and more attention was required by patients who needed specific therapy. It was observed that TCMR was more frequent in all groups of transplanted patients with LD and for patients from G1 who received a kidney from a DD, but graft losses were more frequent in patients who experienced AMR transplanted with DF. These data are in agreement with other publications [21, 23, 34]. Graft loss was observed in patients from G3 who received a kidney from an LD, but no graft loss was demonstrated in immunotherapy patients from G3 and G4 who received a kidney from a DD. These patients also had a better outcome, showing the immunological benefit of immunotherapy for high-risk patients. Better graft survival was observed in G3 in comparison with G1 or G2 for both types of donors. In G3 and G4 patients who received immunotherapy, there was no observed increase in infection.

An ultra-long-term graft survival (42-year) was achieved in a patient transplanted without immunotherapy who received a kidney from his haploidentical mother. The graft was maintained with good function without rejection episodes or nephrotoxicity using monotherapy with azathioprine [33], showing that the type of donor and recipient care and continuous therapy had great influence on this long-term graft survival. This finding opens the discussion about the need for immunotherapy for all recipients, including nonsensitized patients, and it also suggests the use of clinical strategies to minimize the doses of drugs used in triple-maintenance immunosuppressive therapy.

## Conclusion

These data showed a clear association between outcome and rejection episodes in the group of patients with different immunological risk profiles who received or not immunotherapy, as well as increased graft survival in patients from G3 and G4 with medium II and high risk for rejection, and also shows improvement of outcomes, graft function, and graft survival in patients treated with immunotherapy. Our findings strongly suggest that immunotherapy should be used for all patients transplanted with kidneys from deceased donors.

## Data Availability

No applicable.

## Abbreviations

AMR: Antibody-mediated rejection
CIT: Cold ischemia time
CKD: Chronic kidney disease
CyA: Cyclosporine A
DD: Deceased donors
DGF: Delayed graft function
DSA: Donor-specific antibody
LD: Living donors
MFF: Mycophenolate mofetil
MFI: Mean fluorescence intensity
rATG: Rabbit thymoglobulin
SPI-SAB: Solid-phase immunoassay–single antigen beads
TAC: Tacrolimus
TCMR: T-cell–mediated rejection
